# Agility MLC transmission optimization in the Monaco treatment planning system

**DOI:** 10.1002/acm2.12399

**Published:** 2018-06-30

**Authors:** Michael Roche, Robert Crane, Marcus Powers, Timothy Crabtree

**Affiliations:** ^1^ The Department of Medical Physics The Townsville Cancer Centre Douglas Queensland Australia

**Keywords:** Agility MLC, MLC transmission, Monaco, TPF, TPS

## Abstract

The Monaco Monte Carlo treatment planning system uses three‐beam model components to achieve accuracy in dose calculation. These components include a virtual source model (VSM), transmission probability filters (TPFs), and an x‐ray voxel Monte Carlo (XVMC) engine to calculate the dose in the patient. The aim of this study was to assess the TPF component of the Monaco TPS and optimize the TPF parameters using measurements from an Elekta linear accelerator with an Agility™ multileaf collimator (MLC). The optimization began with all TPF parameters set to their default value. The function of each TPF parameter was characterized and a value was selected that best replicated measurements with the Agility™ MLC. Both vendor provided fields and a set of additional test fields were used to create a rigorous systematic process, which can be used to optimize the TPF parameters. It was found that adjustment of the TPF parameters based on this process resulted in improved point dose measurements and improved 3D gamma analysis pass rates with Octavius 4D. All plans calculated with the optimized beam model had a gamma pass rate of > 95% using criteria of 2% global dose/2 mm distance‐to‐agreement, while some plans calculated with the default beam model had pass rates as low as 88.4%. For measured point doses, the improvement was particularly noticeable in the low‐dose regions of the clinical plans. In these regions, the average difference from the planned dose reduced from 4.4 ± 4.5% to 0.9 ± 2.7% with a coverage factor (*k* = 2) using the optimized beam model. A step‐by‐step optimization guide is provided at the end of this study to assist in the optimization of the TPF parameters in the Monaco TPS. Although it is possible to achieve good clinical results by randomly selecting TPF parameter values, it is recommended that the optimization process outlined in this study is followed so that the transmission through the TPF is characterized appropriately.

## 1 INTRODUCTION

In modern radiotherapy, treatment planning systems (TPSs) are used to generate dose distributions with the aim of maximizing tumor control and minimizing normal tissue complications. Traditional forward based treatment planning has been supplemented by inverse planning, which uses dose optimization techniques including intensity modulated radiotherapy (IMRT)^1^ and volumetric modulated arc therapy (VMAT),^2^, ^3^ to satisfy user specified criteria. To achieve the appropriate target coverage and respect the dose constraint criteria for organs at risk, both IMRT and VMAT use many irregularly shaped fields defined by multileaf collimators (MLCs). MLCs have been routinely used in radiotherapy over the past 20 years.^4^, ^5^ Desirable MLC design characteristics include low intraleaf and interleaf transmission, a small tongue and groove effect, a small leaf width, accurate and fast leaf positioning, and most importantly, reproducibility. Reproducibility is paramount in an MLC system as this attribute allows for accurate characterization of the MLCs in the TPS, which in turn facilitates accurate IMRT and VMAT deliveries.

The Monaco 5.11.01 Monte Carlo (MC) treatment planning system (IMPAC Medical Systems, Inc., Maryland Heights, MO (a subsidiary of Elekta AB, Stockholm, Sweden)) uses three‐beam model components to achieve accuracy in dose calculation. First, the linear accelerator photon beam is approximated using a virtual source model (VSM) consisting of a primary photon source, a scatter photon source, and an electron contamination source.^6^, ^7^, ^8^, ^9^ The VSM is used instead of MC transport through the components of the linear accelerator to speed up the calculation. Second, the primary collimator, jaws, and MLC are modeled using transmission probability filters (TPFs).^6^, ^7^, ^8^, ^9^ Similar to the VSM, the TPFs are used instead of direct MC simulation to significantly reduce calculation times. Finally, x‐ray voxel Monte Carlo (XVMC)^10^ is used to calculate the dose in the patient model defined by the patient CT dataset.

This study concentrates on the optimization of the TPF using measurements from an Elekta linear accelerator with an Agility™ MLC (Elekta AB, Stockholm, Sweden).^11^, ^12^ To aid with the optimization of the TPF, several predesigned fields known as the ExpressQA package^13^ have been provided by the vendor. Although these fields can aid with the optimization, a set of additional test fields are recommended in this study which will simplify and improve the TPF optimization process. The optimization of the TPF for the Agility™ MLC has been previously described^14^ and an alternative “potential recipe for MLC modeling” was recommended. However, in this study, no specific details were provided on the purpose of a number of the TPF parameters defined in the Monaco TPS. It was also suggested that certain TPF parameters can be unrealistically adjusted. Point dose measurements and gamma analysis showed that this method provides adequate clinical results; however, setting unrealistic values for TPF parameters is not optimal. This study endeavors to identify an improved TPF optimization process where each TPF parameter can be optimized resulting in a simplified post modeling optimization process. Using this method, the fundamentals of the transmission modeling can be guaranteed, allowing for confidence in the all aspect of the TPF transmission characterization.

## 2 MATERIALS AND METHODS

**Figure 1:**
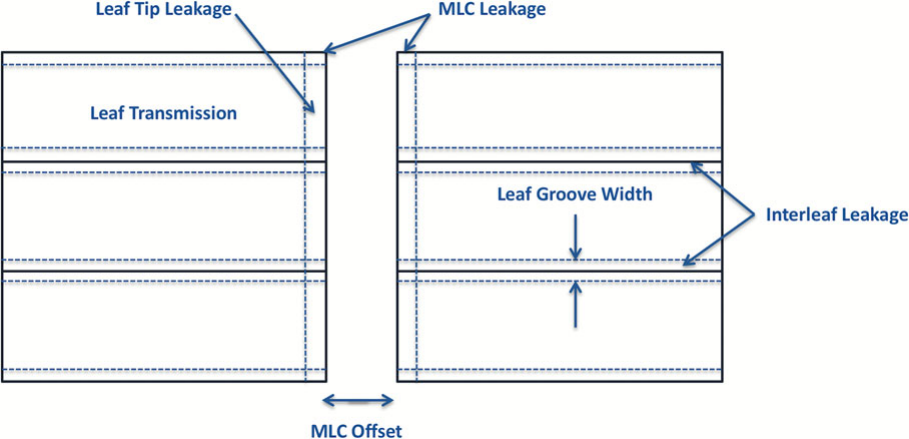
A 2D representation of the geometry of the MLC TPF in the Monaco TPS, including a selection of TPF parameters. Leaf Transmission defines the fractional transmission through an MLC; Leaf Groove Width defines the extent of the leaf groove region outside the MLC; Interleaf Leakage and Leaf Transmission define the increase in transmission between adjacent leaves; Leaf Tip Leakage and Leaf Transmission define the increase in transmission due to the curvature of the MLC tips; MLC Leakage, Interleaf Leakage and Leaf Transmission define the increase in transmission at the corner of the MLC tips; and MLC Offset defines the difference between the prescribed leaf position and the actual value used for dose calculation.

The TPFs in the Monaco TPS are characterized by both geometry and the probability of particle transmission. For the primary collimator, a parameter is set in the vendor modeling process to establish the angle beyond which photons and electrons are attenuated by 99%.^9^ This parameter is not editable by the user from the Monaco TPS. However, for the secondary collimators, the TPFs are editable through several parameters^13^ used to define the transmission probabilities through various regions of the beam modifiers. Editing a number of these transmission parameters appropriately can help differentiate the variation in transmission through the distinct MLC regions. This includes the transmission through the body of the MLCs, between adjacent MLCs and through the MLC tips. Fig. 1 illustrates a 2D representation of the MLC TPF and identifies the various MLC regions. In reality, the TPF is three‐dimensional where the leaf transmission determines its thickness. This thickness is then divided into 11 equally spaced transmission planes so that the transmission of oblique photons can be calculated more accurately.^9^ The TJaw transmission and TJaw Tip Leakage TPF parameters are used to determine the transmission through the jaws that travel transverse to the direction of leaf motion.

Table 1 displays the TPF parameters investigated in the optimization process. More TPF parameters exist in the Monaco TPS; however, they were either for adjusting the position of the collimator planes in the VSM or for increasing the backscatter from the collimators. Adjustments to these TPF parameters were not investigated. Table 1 also displays four MLC models with varying parameters. The “Default” model summarizes the TPF parameters when set to their default value and Model A summarizes the TPF parameters post completion of the optimization process. Two additional models, Model B and Model C, are included to specifically demonstrate the effects of adjusting the MLC Leakage and Leaf Groove Width TPF parameters. Model B is identical to Model A except the MLC Leakage TPF parameter has been adjusted and Model C is identical to Model A except the Leaf Groove Width TPF parameter has been adjusted.

**Table 1:** TPF parameters for various TPS models used in this study

MLC TPF Parameter	Default	Model A	Model B	Model C
Static Leaf Gap (mm)	0.10	0.10	0.10	0.10
Leaf Transmission	0.0030	*0.0032*	*0.0032*	*0.0032*
Inter Leaf Leakage	3.0	*7.0*	*7.0*	*7.0*
TJaw Transmission	0.0050	*0.0032*	*0.0032*	*0.0032*
TJaw Tip Leakage	1.03	1.03	1.03	1.03
MLC Offset (mm)	0.00	*−0.05*	*−0.05*	*−0.05*
Leaf Tip Leakage	1.10	*1.18*	*1.18*	*1.18*
MLC Leakage	0.00	0.00	*0.10*	0.00
Leaf Groove Width (mm)	0.40	0.40	0.40	*1.1*

Underlined italics indicate TPF parameters that have been adjusted from their default value.

### 2.A TPF Optimization

The optimization began with all TPF parameters set to their default value. The function of each parameter was investigated and a value was selected that best replicated measurements with the Agility™ MLC. A number of the TPF parameters are linked; as a result, the parameters were iteratively adjusted throughout the optimization process to improve the agreement between measurements and TPS calculations. The number of iterations required in the optimization process is significantly dependent on the users experience with the TPF in Monaco. An in‐experienced user will most likely have to modify each TPF parameter individually, resulting in a significant number of iterations before the optimal TPF parameters are determined. More experienced user may be able to modify multiple parameters at once, reducing the number of iterations required.

Both the vendor provided fields and a set of additional test fields were used throughout the optimization. TPF parameters were not modified if their default value was determined to be appropriate. All measurements were taken at a gantry angle of 0° and a collimator angle of 0°, while all fields were calculated in Monaco with a 1 mm grid size and a statistical uncertainty of 0.25% per control point. The IEC 61217 geometrical convention is used throughout this paper.

#### 2.A.1 Minimum Leaf Gap

The minimum leaf gap or closed leaf gap is defined as the minimum allowable separation between opposing leaves. For the Agility™ MLC, this should be 1 mm at the leaf bank plane.^12^ The first step in the MLC optimization process is to check the closed leaf gap on the linear accelerator. This was done with a feeler gauge, following the recommended vendor procedure.^15^ Next, the transmission through the closed leaf gap was measured at isocenter, on central axis, and at multiple positions off axis, with Gafchromic EBT3 film (Ashland Specialty Products, NJ). A source‐to‐detector distance (SDD) of 100.0 cm was used for all measurements; and 5 cm of Solid Water^®^ Model 457 (Gammex, WI) was placed on top. Results were normalized to the output on central axis of a 10 × 10 cm^2^ field, measured under the same conditions. Measurements were then compared to transmission values calculated in the Monaco TPS and the effect of modifying the Static Leaf Gap TPF parameter was investigated.

#### 2.A.2 Secondary Collimator Transmission

Once the closed leaf gap was set on the linear accelerator, MLC transmission and diaphragm transmission measurements were performed. To measure the leaf bank transmission, the MLCs were closed at 15 cm off axis and a point dose was measured on central axis. To differentiate the intraleaf and interleaf transmission, a high‐resolution profile was measured perpendicular to the direction of MLC travel. The Y diaphragm transmission was measured at a point on central axis with the diaphragms closed off axis at −12.5 cm and with the MLCs parked behind the thickest section of the diaphragms.^11^

Point dose measurements were taken in an MP3 water phantom (PTW, Freiburg, Germany) using both an FC65‐G Farmer type ionization chamber (IBA, Schwarzenbruck, Germany) and a PinPoint 31014 ionization chamber (PTW, Freiburg, Germany). The long axes of the chambers were placed perpendicular to the direction of leaf motion. Relative measurements of the interleaf transmission were performed with Gafchromic EBT3 film in Solid Water^®^. All measurements were taken with an SDD of 100 cm, at a depth of 5 cm and were normalized to the output on central axis of a 10 × 10 cm^2^ field under the same conditions. Measurements were compared to transmission values calculated in the Monaco TPS and the Leaf Transmission, TJaw Transmission, and Interleaf Leakage TPF parameters were adjusted to match the measured values.

#### 2.A.3 Leaf Offset

The next step in the optimization process is to define the Leaf Offset TPF parameter. The Leaf Offset is described as the difference between the prescribed leaf position, and the actual value used for dose calculation and should be adjusted to match the machine‐specific MLC calibration.^13^ To identify an appropriate value for the Leaf Offset, measurements were performed with a Gafchromic EBT3 film in Solid Water^®^ using the 3ABUT vendor provided predesigned field. All measurements were taken at an SDD of 100 cm, at 5 cm depth. The dose at the junctions formed by the leaf tips of each segment was matched to that calculated in the Monaco TPS by adjusting the Leaf Tip Leakage and Leaf Offset TPF parameters. The effect of adjusting the MLC Leakage parameter was also investigated.

#### 2.A.4 Dosimetric Leaf Gap

The dosimetric leaf gap (DLG) has been described as the difference between the nominal field width defined by the MLC leaves and the full width half maximum (FWHM) of the dose profile, measured parallel to the direction of leaf motion.^16^, ^17^, ^18^ It is possible to measure the DLG using an integral dose method which relates the width of the nominal MLC field to the integral dose of its profile.^16^ To measure the DLG of the Agility™ MLC, five sliding window fields with fixed widths from 15 mm to 4 mm were delivered. Point dose measurements were taken in an MP3 water phantom using both an FC65‐G Farmer type ionization chamber and a PinPoint 31014 ionization chamber. All measurements were taken at an SDD of 100 cm, at a depth of 5 cm and were normalized to the output on central axis of a 10 × 10 cm^2^ field under the same conditions. Transmission through the MLCs contributing to the measured dose was subtracted for each sliding window field using Eq. (1),

(1) $$S_{cp,sw}^{\mathrm{corr}}=S_{cp,sw}\cdot MLCT\times \left(1-\left(w/L\right)\right)$$

where $S_{cp,sw}^{\mathrm{corr}}$ is the corrected sliding window total scatter factor, $S_{cp,sw}$ is the uncorrected sliding window total scatter factor, *MLCT* is the MLC leaf bank transmission measured in section 2.A.2, *w* is the sliding window width, and *L* is the length of the dynamic field, which in this case was 100 mm. The dose under a 10 mm and a 20 mm sliding window field, measured under the same conditions, was also matched to that calculated in the Monaco TPS. This was possible by adjusting the Leaf Tip Leakage and MLC Offset TPF parameters.

#### 2.A.5 Tongue and Groove Effect

The Agility™ MLCs have no tongue or groove, the leaf sides are flat with a constant gap of 90 μm between adjacent leaves.^11^ To reduce interleaf transmission, these gaps are defocused from the x‐ray source with the introduction of an angle in the Agility™ MLCs, creating an effective tongue and groove. Two in‐house fields were created to determine the effective tongue and groove effect of the Agility™ MLCs. The field shapes in Fig. 6(a) and 6(b) were created so that the transmission through the effective tongue and groove region could be determined and compared for (a) beamlets of varying size and (b) under a range of leaves extended into the field at multiple locations. These two fields along with the vendor provided FOURL field (Fig. 6(c)) were used to aid in the optimization of the Leaf Groove Width TPF parameter. All measurements were taken at an SDD of 100 cm, at a depth of 5 cm in Solid Water^®^ with Gafchromic EBT3 film. Measurements were compared to values calculated in the Monaco TPS under the same conditions and the effect of adjusting the Leaf Groove Width parameters was investigated.

### 2.B Validation of TPF Optimization

To validate the TPF optimization, measured point doses and 3D dose matrices for a number of clinical IMRT and VMAT plans were compared to those calculated in the TPS. Point dose measurements were made in the IMRT Matrix Phantom T40026 (PTW, Freiburg, Germany) using a 0.125 cc Semiflex 31010 ionization chamber (PTW, Freiburg, Germany). In total, 30 point dose measurements were made using 10 IMRT and VMAT plans created for various anatomical sites. In the TPS, the dose grid resolution was set to 2 mm and the statistical uncertainty was set to 3.0% per control point. The ESTRO recommended confidence limit of ±3% for ion chamber measurements^19^ was used to identify passing points and measurements were compared to calculations with the default and optimized beam models. Point doses were divided into two categories; low dose and high dose. A low‐dose region was considered to be any region with a dose lower than 50% of the maximum planned dose and a high‐dose region was considered to be any region with a dose greater than 50%.

3D dose matrices were reconstructed with Octavius^®^ 4D (PTW, Freiburg, Germany) from measurements with the Octavius^®^ 1500 detector T10044 (PTW, Freiburg, Germany). The array was placed in the Octavius^®^ 4D rotational phantom and all fields were delivered with the planned gantry angles. The Octavius^®^ 4D rotational phantom was modeled in the TPS as a cylindrical phantom with a uniform density using the CT dataset supplied by PTW. The relative electron density (RED) of the Octavius^®^ phantom was set to 1.016. The statistical uncertainty and dose grid size were set to 3.0% per control point and 2 mm, respectively, for all dose calculations. Dose distributions were analyzed using VeriSoft v.7.0.1.30 (PTW, Freiburg, Germany) using a gamma^20^ criteria of both 3% global dose, 3 mm distance‐to‐agreement, and 2% global dose, 2 mm distance‐to‐agreement where global dose is defined as the maximum dose in the entire analyzed volume. Again, measurements were compared to calculations with both the default and optimized beam models. Gamma analysis results for the 3D dose volume greater than 50% of the maximum delivered dose are displayed.

## 3 RESULTS AND DISCUSSION

**Figure 2:**
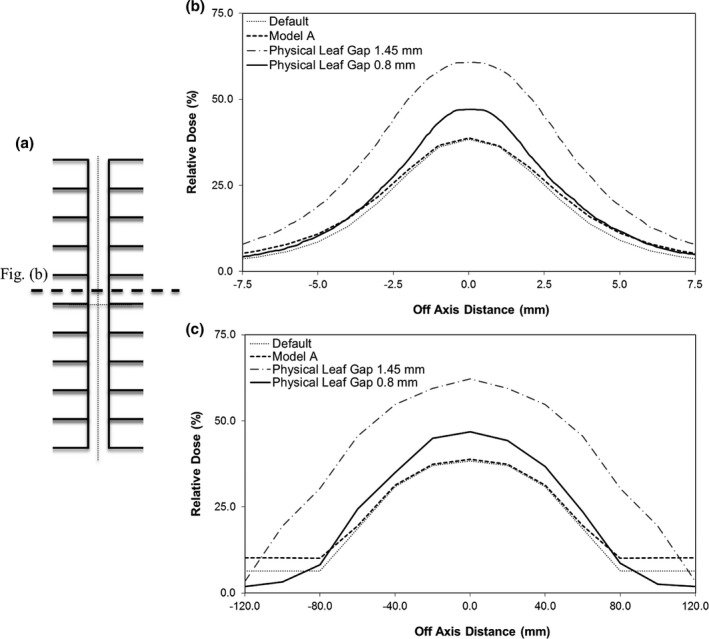
Transmission through the closed leaf gap compared to the Monaco TPS: (a) MLC positioning for the closed leaf gap field, (b) profiles of the transmission through the closed leaf gap at isocenter, and (c) variation in the transmission through the closed leaf gap with off axis distance.

Due to the many small beamlets created by the Monaco TPS in complex IMRT plans, it is important that the closed leaf gap on the linear accelerator is appropriately set and that the TPS correctly models this behavior. Initially, the closed leaf gap measured with the feeler gauge was 1.45 ± 0.04 mm (*k* = 2) at the leaf bank plane. The physical leaf gap was reduced to 0.8 ± 0.04 mm (*k* = 2) so that the measured transmission matched the Monaco TPS. The closed leaf gap was not reduced below 0.8 mm due to the increased likelihood of MLC collisions. As shown in Fig. 2(b), which illustrates the relative transmission through the closed leaf gap at isocenter, even with the new closed leaf gap the Monaco TPS underestimated the transmission under the closed leaves by nearly 20%. The Static Leaf Gap TPF parameter was increased from its default value of 0.1 mm; however, no change in the transmission under the closed leaves was observed. This is because the Static Leaf Gap parameter is used by the Monaco Static MLC Sequencer (SS) and does not affect the transmission through the TPF.^21^ Although the closed leaf gap does not physically change as the MLCs move off axis, the radiation transmission through the closed leaf gap does. This is due to the leaf design which results in the reduction of the transmission through the closed leaf gap as illustrated in Fig. 2(c). The Monaco TPS successfully modeled the reduction in the radiation transmission when the closed leaf gap is moved off axis. However, if the SS does not physically close the MLCs, it is likely that the TPS will overestimate the transmission off axis under very small leaf gaps. No clinically significant difference in modeling the closed leaf gap was identified between the default and optimized beam model.

**Figure 3:**
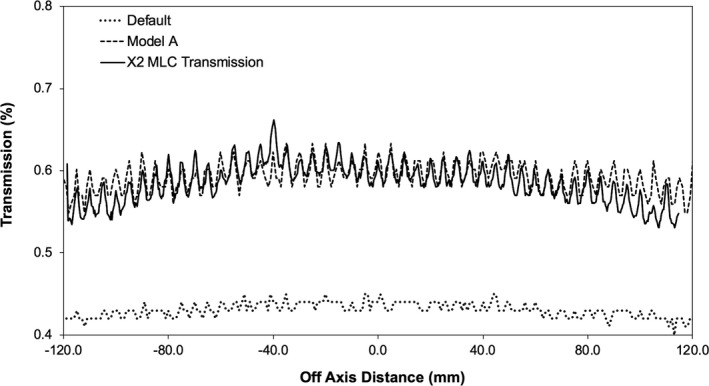
Leaf transmission profile measured perpendicular to the direction of leaf travel, compared to profiles calculated in the Monaco TPS.

Fig. 3 illustrates the measured MLC transmission compared to that calculated in the Monaco TPS. The transmission under the X1 and X2 leaf bank was measured to be 0.60 ± 0.02% (*k* = 2) with the FC65‐G ionization chamber and 0.58 ± 0.02% (*k* = 2) with the PinPoint ionization chamber. The transmission measured with the FC65‐G ionization chamber was higher as the 23.1 mm length of the sensitive volume sampled both the intraleaf and interleaf transmission. The Leaf Transmission and Interleaf Leakage TPF parameters were iteratively adjusted to match the film and ionization chamber measurements. The transmission under the Y1 and Y2 jaw were measured to be 0.38% ± 0.02% (*k* = 2) and the TJaw Transmission TPF parameter was adjusted to match the measurement. It should be noted that the TPF does not modify particle energies for both the MLC and jaw transmission. As a result, when the individual TPF parameters are specified using the above methodology, the combined transmission is not truly the product of the two filters. Adjustments of the TJaw Transmission, Leaf Transmission and Interleaf Leakage TPF parameters were not required for the remainder of the optimization process as the calculated transmission did not change significantly while adjusting other TPF parameters.

**Figure 4:**
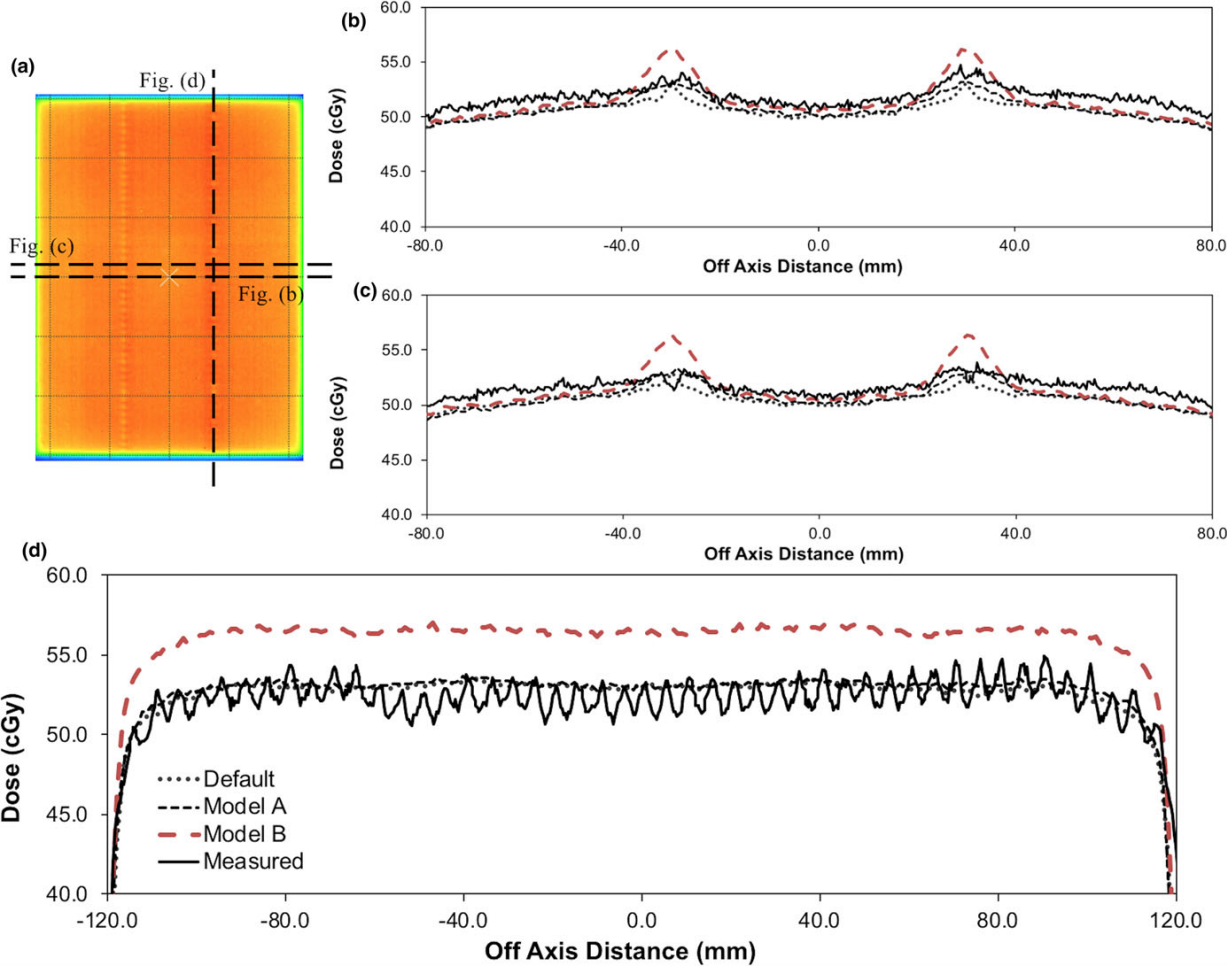
Dose distributions calculated and measured with the 3ABUT vendor test beam: (a) measured dose distribution, (b)–(d) profiles from the relevant dashed lines in (a). The data labels in (d) apply to (b) and (c).

**Figure 5:**
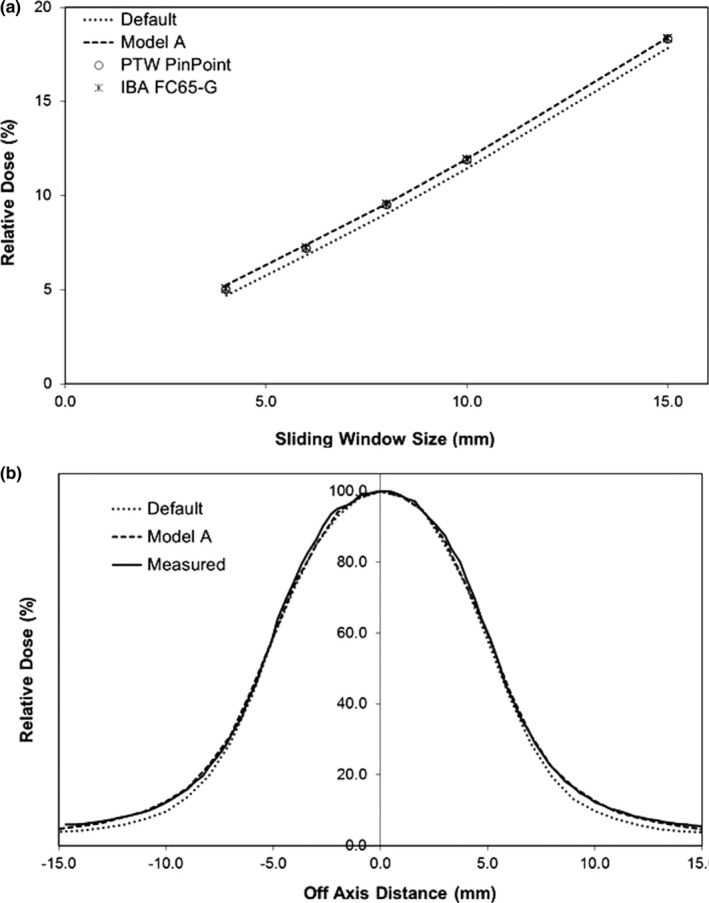
Relative dose distributions resulting from DLG measurements and calculations: (a) integral dose for sliding window fields of decreasing window width, (b) profile taken parallel to the direction of MLC motion under the 1 cm sliding window field, static at isocenter.

The MLC Offset and Leaf Tip Leakage TPF parameters were then iteratively adjusted to match the measured and calculated DLG and vendor provided 3ABUT field. Profile measurements through various regions of the 3ABUT field are illustrated in Fig. 4, while the results from the DLG measurements are illustrated in Fig. 5. Fig. 5(a) illustrates the integral dose for each sliding window decreasing linearly with a reduction in the gap width. Extrapolating a linear regression fit of the data reveals the gap width corresponding to zero charge measurement. Using this methodology, the DLG was measured to be 0.01 ± 0.18 mm (*k* = 2) and 0.03 ± 0.16 mm (*k* = 2) with the FC65‐G and Pinpoint ionization chambers respectively. However, when the sliding window fields were calculated in the TPS with the default model, a DLG of 0.30 ± 0.08 mm (*k* = 2) was calculated. To investigate this discrepancy, the 1 cm sliding window field was measured under static conditions at isocenter. A profile from this measurement, parallel to the direction of MLC motion, is illustrated in Fig. 5(b). The default model underestimated the dose for the static field off axis between 7.5 cm to 15.0 cm. To correct this in the TPS, the Leaf Tip Leakage TPF parameter was increased. At the same time, the MLC Offset TPF parameter was reduced to ensure a continued match with the 3ABUT field. Figs. 4(b), 4(c), and 4(d) show measured and calculated profiles through the 3ABUT field. As illustrated by the results of Model A, the measured leaf end corner transmission was not calculated appropriately by the TPS for the fully optimized model. This is because no adjustments were made to the MLC Leakage TPF parameter. Figures 4(b), 4(c) and 4(d) also show the results of adjusting the MLC Leakage TPF parameter to 0.10 (Model B). A sharp increase in the dose in the MLC tip region is illustrated, with a negligible improvement in the calculation of leaf end corner transmission. These results were attributed to the relatively large size of the MLC Leakage region in the TPF ((0.5 mm × 2.0 mm) × 2) and as a result, adjustments to the MLC Leakage parameter are not recommended. To replicate the leaf end corner leakage of the Agility™ MLC with the current TPF, the MLC Leakage parameter would have to be increased further and the MLC Offset parameter decreased significantly. It is expected that replicating the leaf end corner leakage in this manner will affect small field output factors and degrade clinical plan results. However, in future versions of Monaco, an adjustment to the size of the MLC Leakage TPF region may allow for increased accuracy in the calculation of the leaf end corner transmission. It is estimated that a MLC Leakage TPF region of 0.5 mm × 1.0 mm to replace the current 0.5 mm × 2.0 mm region would be sufficient to aid with modeling the leaf end corner transmission on the Agility™ MLC.

**Figure 6:**
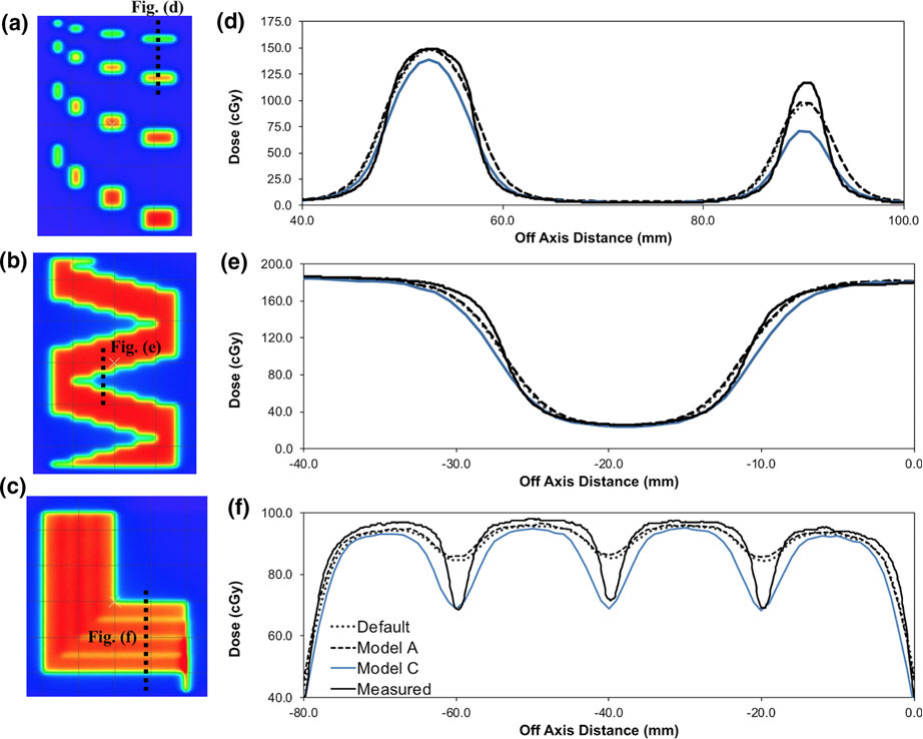
In‐house and vendor provided fields created to determine the effective tongue and groove of the Agility™ MLCs: (a)–(b) measured dose distribution of in‐house created fields, (c) measured dose distribution of vendor provided FOURL field, (d) profiles from the relevant dashed lines in (a), (e) profiles from the relevant dashed lines in (b), and (f) profiles from the relevant dashed lines in (c). The data labels in (f) apply to (d) and (e).

Fig. 6 illustrates the two in‐house fields along with the vendor provided FOURL field used to aid in the optimization of the Leaf Groove Width TPF parameter. A selection of dose distributions resulting from the three test fields are also displayed in Fig. 6(d)–(f). The Leaf Groove Width TPF parameter was adjusted from its default value of 0.40 to 1.1 to match the dose at the effective tongue and groove junctions of the vendor provided FOURL field. Model C in Figs. 6(d), 6(e) and 6(f) illustrates the results of this adjustment. Fig. 6(d) shows a reduction in dose of approximately 7% under the 1 cm × 3 cm beamlet, while the reduction in dose under the 0.5 cm × 3 cm beamlet was approximately 30%. This reduction in dose is not desirable and is due to a decrease in the width of the beamlets as the Leaf Groove Width parameter is increased. Similar reductions in dose were measured under the 1 cm × 2 cm and 0.5 cm × 2 cm beamlets. Fig. 6(e) illustrates the agreement of the TPS with measurements across the effective tongue and groove of the Agility™ MLC. None of the TPS models had the ability to model the effective tongue and groove accurately; however, Model C produced the poorest results. As a result, during the optimization of the TPF no adjustment to the Leaf Groove Width TPF parameter is recommended. It is also recommended that the vendor provided FOURL field is not used to match the dose at the effective tongue and groove junctions. The limited accuracy of the TPF in replicating the effective tongue and groove of the Agility™ MLC was noted. A modification to the modeling of the transmission in the TPF through the tongue and groove region is required to create better agreement with measured transmission on the Agility™ MLC. This modification will likely require the introduction of additional TPF parameters to model the transmission in this region.

**Figure 7:**
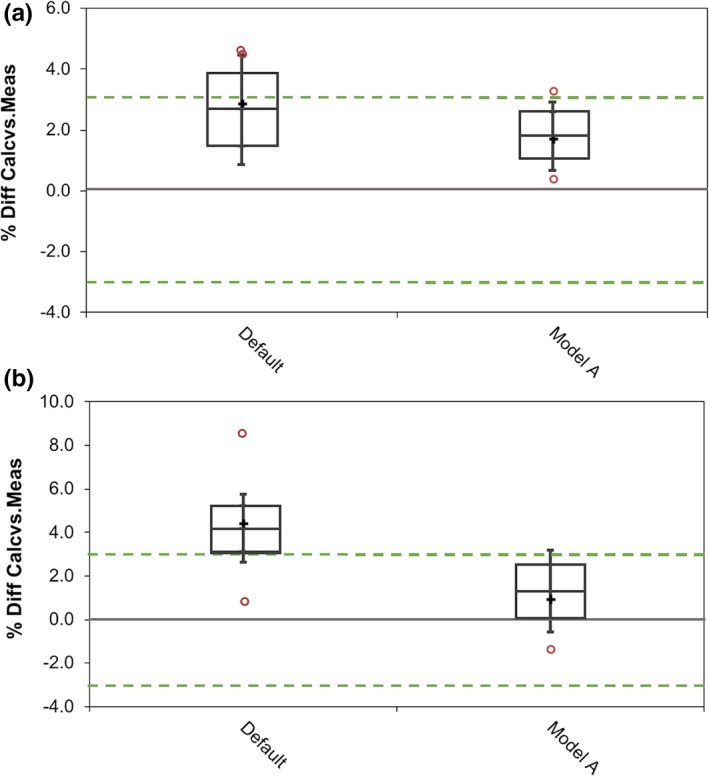
Whisker and box plots of the difference between calculated and measured point doses (a) high‐dose region and (b) low‐dose region. “+” illustrates the mean, the inner quartile range (IQR) is illustrated by the boxes, and the whiskers illustrate the 1.5*IQR. Open circles are used to display any points outside 1.5*IQR. The TPF parameters for each model are displayed in Table 1.

**Figure 8:**
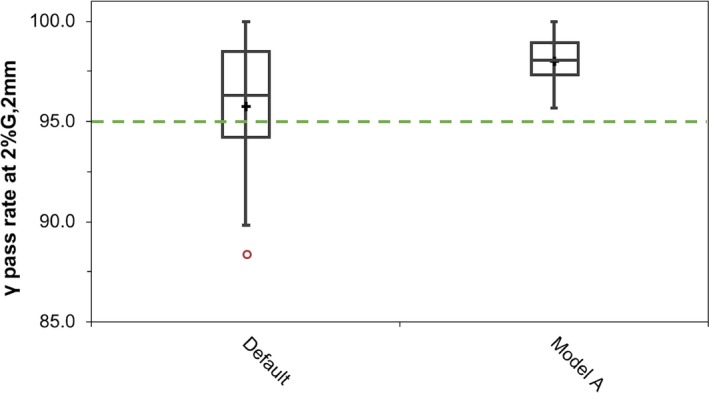
Whisker and box plots of the gamma pass rates (2% global dose/2 mm DTA) of the plans for the default and optimized beam model. “+” illustrates the mean, the inner quartile range (IQR) is illustrated by the boxes, and the whiskers illustrate the 1.5*IQR. Open circles are used to display any points outside 1.5*IQR. The TPF parameters for each model are displayed in Table 1.

Once the final optimized model was created, measured point doses and 3D dose matrices for a number of clinical IMRT and VMAT plans were compared to those calculated in the TPS. Figs. 7(a) and 7(b) illustrate the point dose measurement results for the clinical plans. A significant improvement in the number of points passing the ESTRO recommended confidence limit of ±3% for ion chamber measurements was recorded with the optimized beam model. This improvement was particularly noticeable in the low‐dose regions of the clinical plans. Fig. 8 illustrates the gamma pass rates for the plans measured with Octavius^®^ 4D using the gamma criteria of 2% global dose, 2 mm DTA with a 10% threshold. All plans calculated with the optimized beam model had a gamma pass rate of >95%, while all plans calculated with the default beam model had lower gamma pass rates. For a gamma criteria of 3%/3 mm, there was an insignificant difference in the pass rates for the default and optimized beam models.

### 3.A TPF optimization process

The below procedure is recommended for the optimization of the TPF in the Monaco TPS,

1. Start the optimization process with the default model.
2. Measure and adjust the closed leaf gap on the Agility™ MLC to match calculations from the Monaco TPS. Be aware that reducing the closed leaf gap below 1 mm (at the leaf bank plane) can increase the likelihood of MLC collisions.
3. Iteratively adjust the Leaf Transmission, TJaw Transmission and Interleaf Leakage TPF parameters to match the measured transmission values.
4. Iteratively adjust the Leaf Tip Leakage and MLC Offset TPF parameters to match the DLG and 3ABUT vendor provided field. The MLC Leakage TPF parameter may be adjusted, if in future versions of Monaco the size of the affected TPF regions are reduced.
5. In the current version of Monaco (5.11.01), no adjustment to the Leaf Groove Width TPF parameter is recommended. An improvement in the ability of the TPF to model the transmission through the tongue and groove region is required before better agreement with the effective tongue and groove on the Agility™ MLC can be obtained.
6. Verify the accuracy of the optimized beam model by creating several different IMRT and VMAT plans for sites that will be treated clinically. Follow a rigorous plan specific quality control procedure.

## 4 CONCLUSION

Throughout this study, the ability of the Monaco TPS to model the transmission through the MLC and jaws of the Agility™ MLC was investigated. Although direct simulation of particles through the beam modifiers would be the most accurate method to achieve this, calculation time limitations currently make this impractical. The use of an optimized TPF to model the transmission has been shown to achieve good clinical results for both IMRT and VMAT treatment techniques. The effect of relevant TPF parameters has been provided along with a set of additional test fields which will simplify and improve the TPF optimization process. Although it is possible to achieve good clinical results by randomly selecting TPF parameter values, it is recommended that the optimization process outlined in this study is followed so that the transmission through the TPF is characterized appropriately. To improve calculation accuracy, potential future revision of the TPF may look at reducing the size of the leaf end corners in the TPF and providing additional TPF parameters so that the effective tongue and groove on Agility™ MLC can be accurately characterized.

## CONFLICT OF INTEREST

No conflicts of interest.
